# Comment on "Genetic variants in miR-146a and miR-196a2 in endometriosis: a Brazilian study"

**DOI:** 10.1590/1806-9282.20241085

**Published:** 2024-12-02

**Authors:** Chunfeng Guo, Xiaoyan Liu

**Affiliations:** 1 Weihai Central Hospital, Department of Gynecology – Weihai, China.

Dear Editor,

We were very pleased to read the study by Oliveira et al.^1^, and his colleagues in which they revealed that genetic variants in miR-146a and miR-196a2 are not implicated in the development of endometriosis. This study provides very valuable insights for improved diagnosis of endometriosis to date. However, some views should be raised in my views.

The first overlooked issue is that the study ignored the effect of age on the results. Some studies show that genetic variation is closely related to age^2^. In this study, the researchers did not provide information about the general demographic characteristics of the study participants in both groups. Therefore, we do not know whether there are differences between the two groups in common confounders such as gender or age.

The discrepancies in relation to the number of cases and controls are due to non-real-time amplification of some samples or the samples are over. Participants’ inclusion in the study occurred in the period from 2012 to 2016. It is obvious that the samples were collected at different times. The storage conditions of samples can affect DNA quality^3^. The storage conditions of DNA collected at different times in this study are unclear.

In summary, confounding factors and sample storage conditions affect experimental results. Future studies should fully consider confounding factors such as age, gender, sample collection time, and storage conditions.
